# Modulation of Tumor Cell Survival, Proliferation, and Differentiation by the Peptide Derived from Tenascin-C: Implication of **β**1-Integrin Activation

**DOI:** 10.1155/2012/647594

**Published:** 2011-12-19

**Authors:** Takuya Iyoda, Fumio Fukai

**Affiliations:** ^1^Department of Molecular Patho-Physiology, Faculty of Pharmaceutical Science, Tokyo University of Science, 2641 Yamazaki, Noda-shi, Chiba 278-8510, Japan; ^2^Center for Drug Delivery Research, Research Institute for Science and Technology, Tokyo University of Science, 2641 Yamazaki, Noda-shi, Chiba 278-8510, Japan

## Abstract

Cell adhesion to extracellular matrix (ECM) participates in various biological processes, such as cell survival, proliferation, differentiation, and migration. Since these processes are essential for keeping homeostasis, aberration of these processes leads to a variety of diseases including cancer. Previously, we found that a peptide derived from tenascin- (TN-) C, termed TNIIIA2, stimulates cell adhesion to ECM through activation of **β**1-integrin. It has been shown that TNIIIA2 can modulate cell proliferation and differentiation. Interestingly, TNIIIA2 could not only enhance cell proliferation but also induce apoptotic cell death, depending on cellular context. In this review, we show the function of the peptide TNIIIA2 in cell survival, proliferation, and differentiation and refer to the possibility of new strategy for tumor suppression by regulating cell adhesion status using the ECM-derived functional peptides.

## 1. Introduction

**Figure 1:**
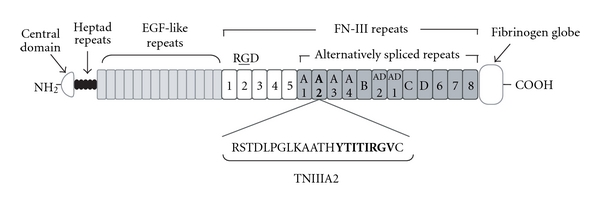
Schematic illustration of tenascin-C domain structure. Sequences analogous to antiadhesive peptide, FNIII14 (YTIYVIAL), are presented in alternative splicing region of TN-C.

Tenascin- (TN-) C, one of extracellular matrix (ECM) proteins, is expressed predominantly during embryogenesis, wound healing, and neoplastic processes. Since TN-C mRNA is alternatively spliced within the fibronectin type III-like (FN-III) repeats (Figure 1), various isoforms of TN-C could be generated. It has been identified that TN-C shows multifunctional properties including effects on cell adhesion, migration, proliferation, survival, and differentiation. Since this ECM protein works as a modulator of cell-matrix interaction but does not seem to contribute directly to the structural elements formation, TN-C is classified as a member of the matricellular protein family. Matricellular proteins regulate cellular function and matrix production through multiple interactions with their cellular receptors, and through modulating expression and activity of cytokines, growth factors, and proteinase [1, 2]. For cell adhesion, the functions of TN-C are particularly complex; the TN-C substrate supports attachment of some cell types but is nonadhesive or even repulsive for other cell types. Various domains of TN-C molecule, including alternative splicing domains, have been implicated in its multifunctional properties. However, the details of their contribution to the adhesion modulatory effects of TN-C are still unclear.

The ECM proteins often harbor functionally active sites within their own molecules. Since these cryptic active sites (matricryptic sites) are disclosed by proteolytic degradation with inflammatory proteinases, the relations between the exposure of matricryptic sites and the development of various diseases have been investigated. We previously found a 22-mer peptide termed FNIII14 from fibronectin (FN), which plays an important role in promoting cell adhesion. FNIII14 strongly suppresses FN-mediated cell adhesion by inhibiting the activation of *α*4*β*1 (VLA-4) and *α*5*β*1 (VLA-5) integrin [3, 4]. It has been determined that the antiadhesive activity of FNIII14 depends on its C-terminal amino acid sequence, YTIYVIAL [3]. We thought that this matricryptic antiadhesive site should be exposed by either FN degradation with matrix metalloproteinase- (MMP-) 2, or FN interaction [5]. Subsequently, we found several sequences similar to the YTIYVIAL sequence of FN in TN-C. Two analogous sequences, YTITIRGV and YTIYLNGD, are present in the FN-III repeat A2 of the alternative splicing region and the C-terminus fibrinogen-globe, respectively (Figure 1). Surprisingly, we observed that a 22-mer TN-C peptide containing YTITIRGV, termed TNIIIA2, stimulates cell adhesion to FN by inducing conformational and functional activation of *β*1-integrin. We also observed that the active site of TNIIIA2, which is also cryptic and exposed by MMP-2 processing, may induce a lateral interaction of *β*1-integrin with the cell surface heparan sulfate proteoglycans (HSPGs), including syndecan-4 ectodomein. Additionally, it has been reported that cytokine-stimulated adhesion via VLA-4 and VLA-5 to FN is rapid (reaching a max within 30 minutes) but transient (returning to basal levels after several hours) [6]. In sharp contrast, TNIIIA2 has the ability to strongly activate *β*1-integrins and to sustain this activated status, probably due to stabilization of the active *β*1-conformation through lateral association with syndecan-4 [7]. Moreover, we observed that TNIIIA2 has a potential to induce apoptotic cell death in nonadherent tumor cells, whereas this peptide also induces aggressive cell growth in nontransformed adherent cells. The evidence from the series of studies with TNIIIA2 shows the possibility that the effect of TN-C in tumor progression has close relation with the behavior of TNIIIA2. In this review, we describe in detail about current knowledge of the effect of TNIIIA2 on various tumor cell phenotypes.

## 2. Host-Beneficial Effects of TNIIIA2 in Hematopoietic Progenitor Cell Types

### 2.1. Induction of Apoptotic Cell Death in Leukemic Cell by TNIIIA2

In ordinary proliferation and survival of hematopoietic stem and progenitor cells, it has been reported that FN plays an important role via the FN-receptors, such as VLA-4 and VLA-5 [8]. Like their normal counterparts, transformed hematopoietic progenitor cells need signals from the FN for their survival and proliferation during their malignant progression [9–11]. This survival effect of FN/ECM interaction is due to prevention of apoptosis [12, 13]. Additionally, increasing evidence has demonstrated that adhesion of hematopoietic tumor cells to FN via VLA-4 and VLA-5 confers a multidrug resistance phenotype, commonly referred as cell adhesion-mediated drug resistance (CAM-DR) [14]. These facts indicate that integrin signal is important for regulating tumor progression.

**Figure 2:**
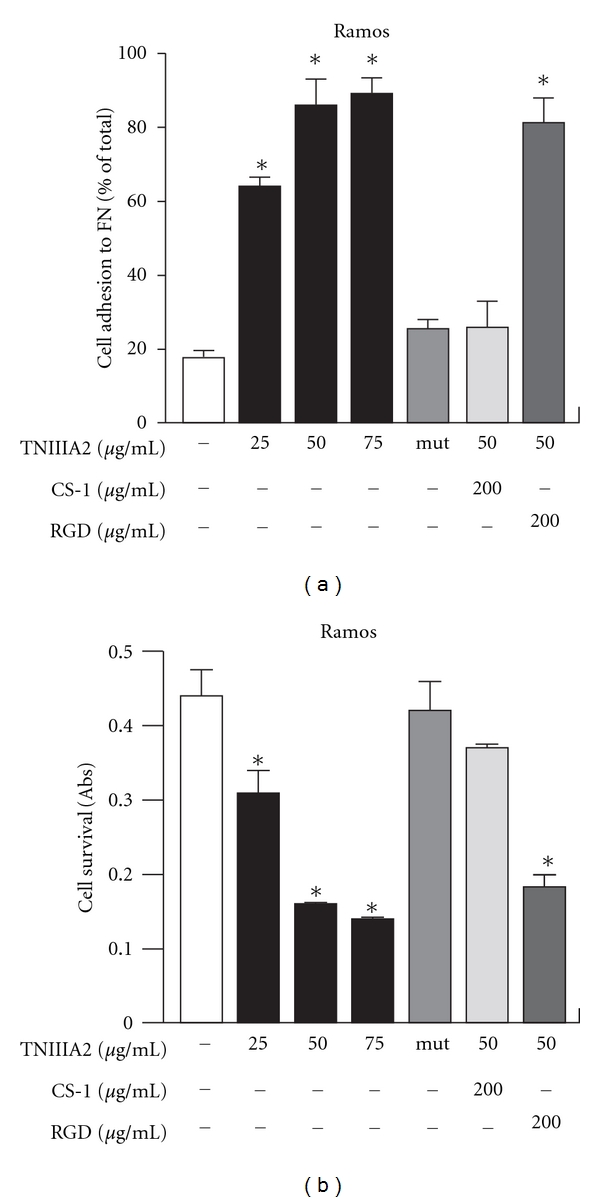
Effect on cell growth of forced adhesion of Ramos cells to FN. (a) shows the results of cell adhesion assay. The percentage of adhere cells are shown relative to the total number of cells seeded into the well. In (b), the effect of induced adhesion to FN on Ramos cell survival was shown. CS-1: connecting segment 1 peptide. (figures were modified from Figure 1 of [19]). *P < 0.05 versus untreated control.

**Figure 3:**
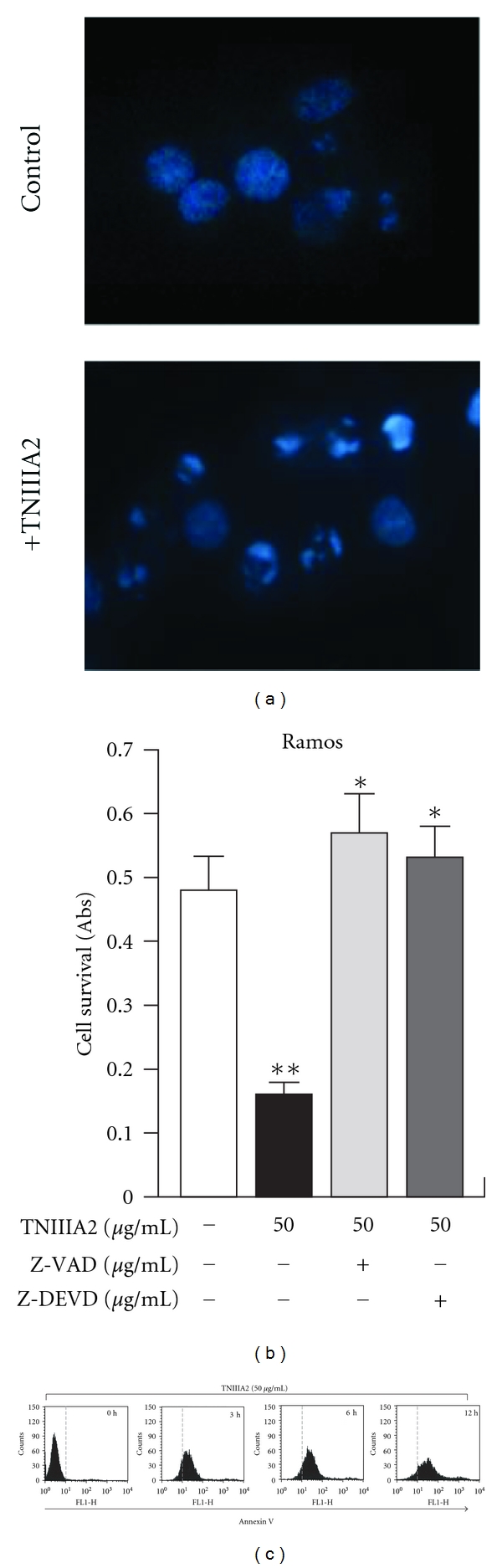
Apoptosis was induced on Ramos cells with TNIIIA2 administration. (a) Hoechst staining of Ramos cells treated with TNIIIA2. (b) Effect of caspase inhibitors on Ramos cell survival, treated with TNIIIA2. (c) Cell surface exposure of phosphatidylserine on Ramos cell treated with TNIIIA2 (figures were modified from Figure 2 of [19]). *P < 0.05 versus TNIIIA2 single treated sample, **P < 0.05 versus untreated control.

Constitutive expression of TN-C has been observed on lymphoid tissues, such as adult bone marrow and lymph nodes [15, 16]. It has also reported that the expression of TN-C is transiently upregulated in pathological states, including inflammation and tumorigenesis [17, 18]. Therefore, lymphoid tissues of patients with hematopoietic malignancy should show highly increased expression of TN-C. Since TNIIIA2 can induce cell adhesion to FN also in hematopoietic tumor cells (Figure 2(a)), it is easily presumed that this peptide may induce enhancement of cell survival and proliferation. However, when hematopoietic tumor cells are forced to adhere to FN substrate by TNIIIA2, these cells undergo apoptotic cell death (Figures 2 and 3). We found that VLA-4 expression is essential for TNIIIA2-induced apoptosis in hematopoietic tumor cell lines. For example, U937 cells, expressing both VLA-4 and VLA-5, underwent apoptosis only when adhered to FN fragments containing the VLA-4-binding sites, and this apoptosis was specifically abrogated by the VLA-4 antagonist, but not by VLA-5 agonist [19]. These results suggest that TNIIIA2-induced forced adhesion to FN via *α*4*β*1 integrin leads to apoptotic cell death in hematopoietic tumor cells.

Our observation seems to be inconsistent with the theory “CAM-DR”. However, there have been also several reports demonstrating the negative effects of cell adhesion on cell survival. Integrin-mediated adhesive interaction with FN was shown to lead apoptosis in myeloid [20, 21] and erythroid progenitor cell lines [22]. To explain this discrepancy, we hypothesized that a moderate adhesion to FN may be favorable for continuous survival in hematopoietic tumor cells. We previously demonstrated that leukemic cell adhesion to bone marrow FN via VLA-4 generated CAM-DR, which could be a major cause of recurrence in acute leukemia patients [23, 24]. Additionally, we recently demonstrated using *in vitro* and *in vivo* experiments that combination therapy with an anticancer drug and antiadhesive peptide, FNIII14, which is capable of inactivating *β*1-integrins, effectively overwhelms the CAM-DR of AML [25]. In a series of previous reports investigating CAM-DR demonstrated that hematopoietic tumor cells show chemoresistancy through spontaneous adhesion to FN without addition of integrin activators [14, 24, 26, 27]. It has been shown that spontaneous adhesion of hematopoietic tumor cells is induced mainly by *β*1-integrin activation through the interaction between cytokine and G protein-coupled receptor (GPCR) [28]. Additionally, it has also been reported that cytokine-stimulated adhesion through VLA-4 and VLA-5 to FN is rapid (reaching a max within 30 minutes) but transient (returning to basal levels after several hours) [6]. Therefore, it appears likely that CAM-DR may be induced through weak or moderate adhesion to FN. In sharp contrast, TNIIIA2 has the ability to strongly activate *β*1-integrins and to sustain this activated status. We suppose that this difference in the state of *β*1-integrin activation should produce the difference in adhesion-induced cellular responses.

How does TNIIIA2 transmit their signal into hematopoietic tumor cells? We previously found that TNIIIA2 requires syndecan-4 as a membrane receptor for activation of *β*1-integrin [7]. Actually, syndecan-4 expression, besides VLA-4, was essential for TNIIIA2-induced apoptosis [19]. Syndecan-4 probably contributes to the sustained activation of VLA-4 through a lateral association with it [7]. Interestingly, TNIIIA2 exhibited no remarkable pro-apoptotic effects on normal peripheral blood cells, such as neutrophils, monocytes, and lymphocytes. It is well known that expression of syndecans is highly regulated with respect to developmental expression and cell-type specificity. Actually, it has been reported that very little syndecan-4 is present on polymorphonuclear leukocytes and peripheral blood mononuclear cells (PBMCs) [29, 30]. Moreover, we tested several hematopoietic tumor cell lines with various expression levels of VLA-4 and syndecan-4 and suggest that syndecan-4 is a key molecule in adhesion-regulated apoptosis induced by TNIIIA2 administration (Table 1).

Although the molecular mechanisms underlying TNIIIA2-induced apoptosis were not defined in detail, these data clearly showed that integrin-mediated adhesion plays a negative role in the survival of hematopoietic progenitor/tumor cells. TNIIIA2 activity embedded in TN-C molecule could contribute, once exposed, to preventing prolonged survival of hematopoietic malignant progenitors. Further study is needed to examine whether the TNIIIA2-related matricryptic site is exposed at its functional level in lymphoid tissues with hematopoietic malignancy.

### 2.2. Acceleration of Erythroid Differentiation by TNIIIA2

Besides hyperproliferation, incomplete differentiation of blood cells is the major phenomena observed in myeloid leukemia. Similar to the proliferation, differentiation of hematopoietic stem and progenitor cells occurs in the bone marrow and fetal liver [8, 31–35]. Although cytokines and growth factors are strong regulator of hematopoiesis, it is generally accepted that the adhesive interactions between hematopoietic stem/progenitor cells and the microenvironment also influence hematopoiesis. Stromal cells of the bone marrow and fetal liver form a hematopoietic microenvironment, called a “niche”. This microenvironment niche plays a pivotal role in the regulation of proliferation and differentiation of hematopoietic stem and progenitor cells. In addition to stromal cells, ECM proteins in lymphoid tissues, such as FN, TN, collagen, laminin, and proteoglycans (PGs), have been implicated as essential components of the microenvironment that regulates hematopoiesis. Among these macromolecules, FN is known as the most important protein of the microenvironment niche in the bone marrow and fetal liver [36–40].

In the case of erythropoiesis, the importance of the cell adhesion of erythroid progenitors to FN via the FN-receptors VLA-4 and VLA-5 has been demonstrated [22, 40–44]. A number of previous studies demonstrated direct adhesion of erythroid progenitor cells to FN. FN functions as an adherent substrate scaffolding erythroid progenitor cells to support their survival and proliferation [22, 40]. Furthermore, it has been postulated that adhesive interaction with FN via FN receptors contributes to the regulation of erythroid differentiation [22, 40–44]. In particular, the importance of VLA-4-mediated adhesion to FN and/or VCAM-1 on stroma cells has been implicated by *in vitro* and *in vivo* studies using antagonist for VLA-4 and VLA-5 [22, 40–44]. However, the substantial role of these FN receptors and their functional assignment in erythroid differentiation were not fully understood.

**Figure 4:**
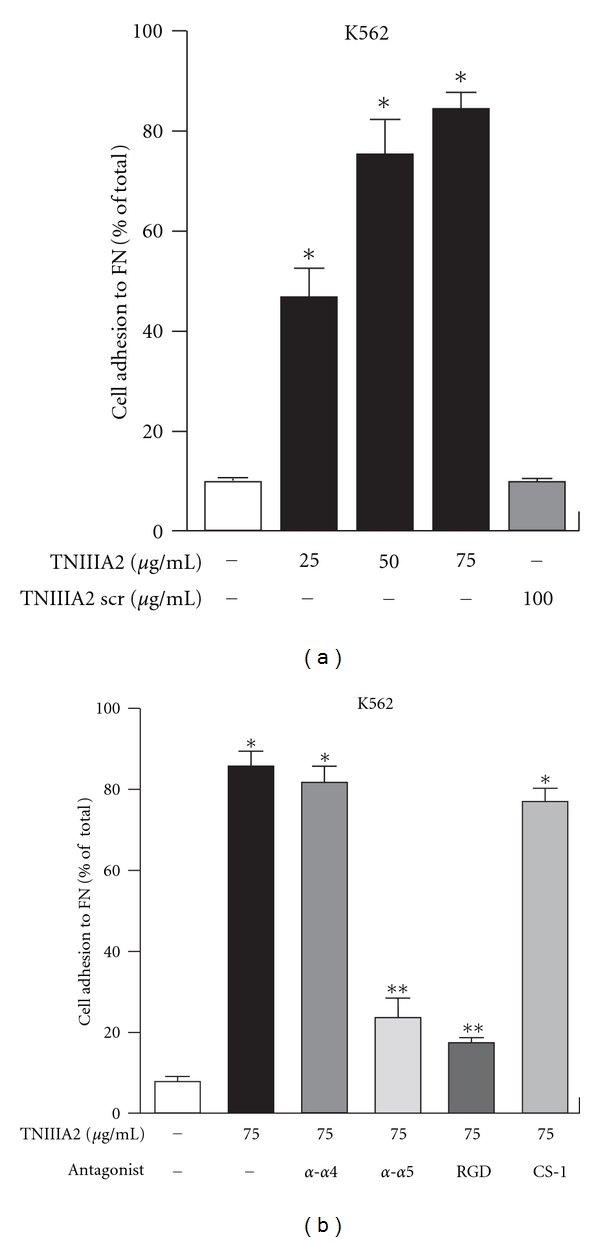
Adhesion of K562 cells to FN through *α*5*β*1 integrin activation. (a) Dose dependency of TNIIIA2-induced adhesion of K562 cells. (b) Effects of antagonist for VLA5 (*α*-*α*5 Ab and RGD) and VLA4 (*α*-*α*4 Ab and CS-1) on TNIIIA2-induced adhesion to FN in K562 cells (figures were modified from Figure 1 of [45]). *P < 0.05 versus untreated control, **P < 0.05 versus TNIIIA2 single treated sample.

**Figure 5:**
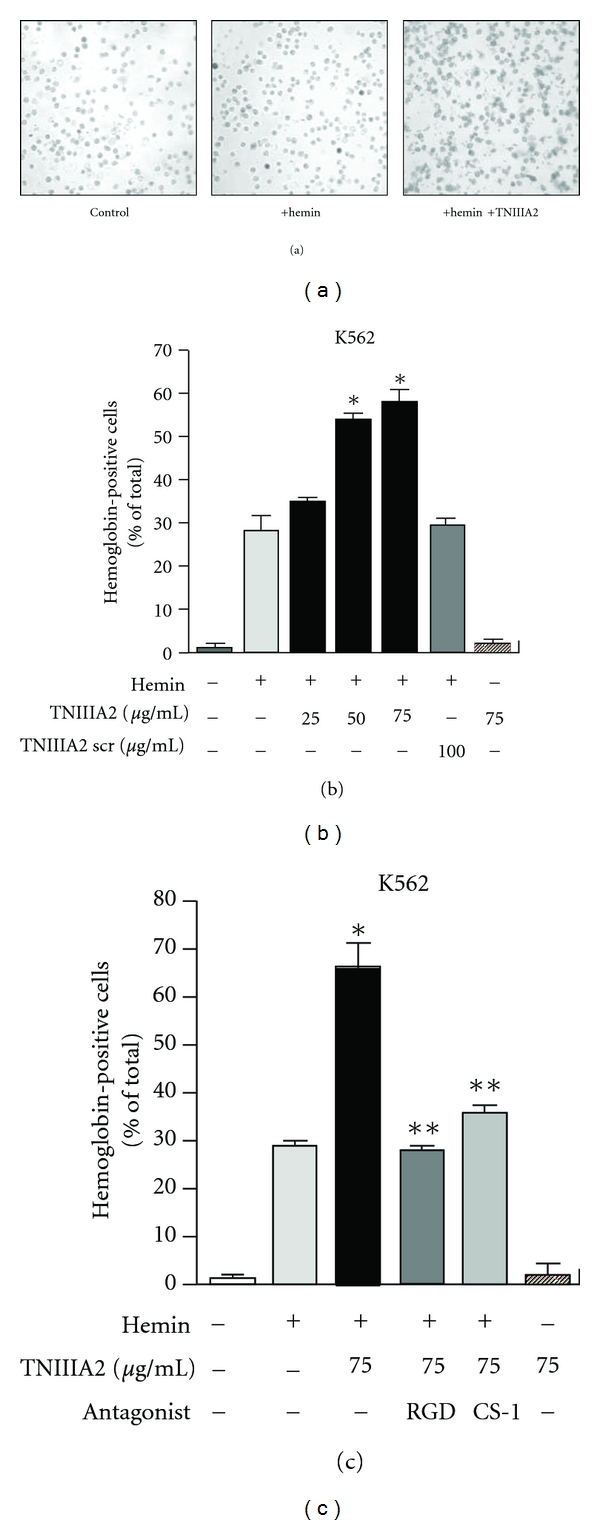
Adhesion dependent acceleration of hemin-induced erythroid differentiation of K562 cells. (a, b) Effect of TNIIIA2 on erythroid differentiation of K562 cells, induced by hemin. Typical image of erythroid differentiation was shown in (a). (c) Effects of antagonist for VLA5 (RGD) and VLA4 (CS-1) on TNIIIA2-induced acceleration of erythroid differentiation (figures were modified from Figures 2(a), 3(a), and 3(c) of [45]). *P < 0.05 versus Hemin single treated sample, **P < 0.05 versus Hemin and TNIIIA2 treated sample.

We recently reported that hemin-induced erythroid differentiation was greatly enhanced when K562 cells were forced to adhere to FN by activating VLA-5 with TNIIIA2 (Figures 4 and 5). Since FN receptor antagonists abrogated the acceleration of erythroid differentiation, the stimulatory effect of TNIIIA2 on erythroid differentiation might be dependent on adhesion of K562 cells to FN (Figures 4, 5(a) and 5(b)). The adhesion-dependent acceleration of hemin-induced erythroid differentiation may be responsible for the VLA-5-mediated adhesion to FN, because K562 cells reportedly express only VLA-5 as the FN receptor [45, 46]. Nevertheless, the stimulatory effect of TNIIIA2 on hemin-induced erythroid differentiation was abrogated not only by a VLA-5 antagonist (RGD peptide) but surprisingly also by a VLA-4 antagonist (CS-1 peptide) (Figure 5(c)). This conflicting result was explained by the observations that forced adhesion to FN resulted in the induction of VLA-4 expression in K562 cells [45].

Several studies demonstrated that activation of p38 and/or JNK but not ERK is required for erythroid differentiation induced by butyrate [47], erythropoietin [48], hydroxyurea [49], or hemin [50], although another study reported the involvement of ERK in erythroid differentiation induced by hemin [51]. We also observed that the phosphorylation of p38, which was shown to play a crucial role in hemin-induced erythroid differentiation and its acceleration by TNIIIA2, was suppressed by antagonists for VLA-4 and -5 [45]. From these observations, we supposed that prolonged adhesion to FN, mediated through VLA-5, induced VLA-4 expression in K562 cells and the resulting adhesive interaction of FN with newly expressed VLA-4 participated in differentiation via phosphorylation/activation of p38 MAP kinase, which was shown to serve as a signaling molecule crucial for hemin-induced erythroid differentiation. It has also been demonstrated that TN-C on bone marrow stromal cells may play an important role in erythropoiesis [52]. As mentioned above, our observations suggest that the peptide derived from TN-C, TNIIIA2, can accelerate hemin-induced erythroid differentiation. TN-C is known to be abundantly expressed in the stromal cells of immune organs including the bone marrow [15, 16] and is susceptible to proteolytic modification [53]. Therefore, it might be possible that inducing the exposure of TNIIIA2 region works as a beneficial therapeutic treatment reducing one of symptoms in tumor, “poor differentiation”.

## 3. Hyperstimulation of Nontransformed Cell Proliferation by TNIIIA2

It is well known that normal adherent cell types, such as fibroblastic and epithelial cells, undergo apoptosis like cell death when the *β*1-integrins of these cells lose the interaction with ECM. This process is termed “anoikis” [54, 55] and plays a fundamental role preventing dissemination of the cells to inappropriate site. It is also well accepted that tumor cells develop anoikis-resistance, resulting in acquisition of metastatic ability. Thus, understanding the mechanisms how tumor cells evade anoikis is important.

**Figure 6:**
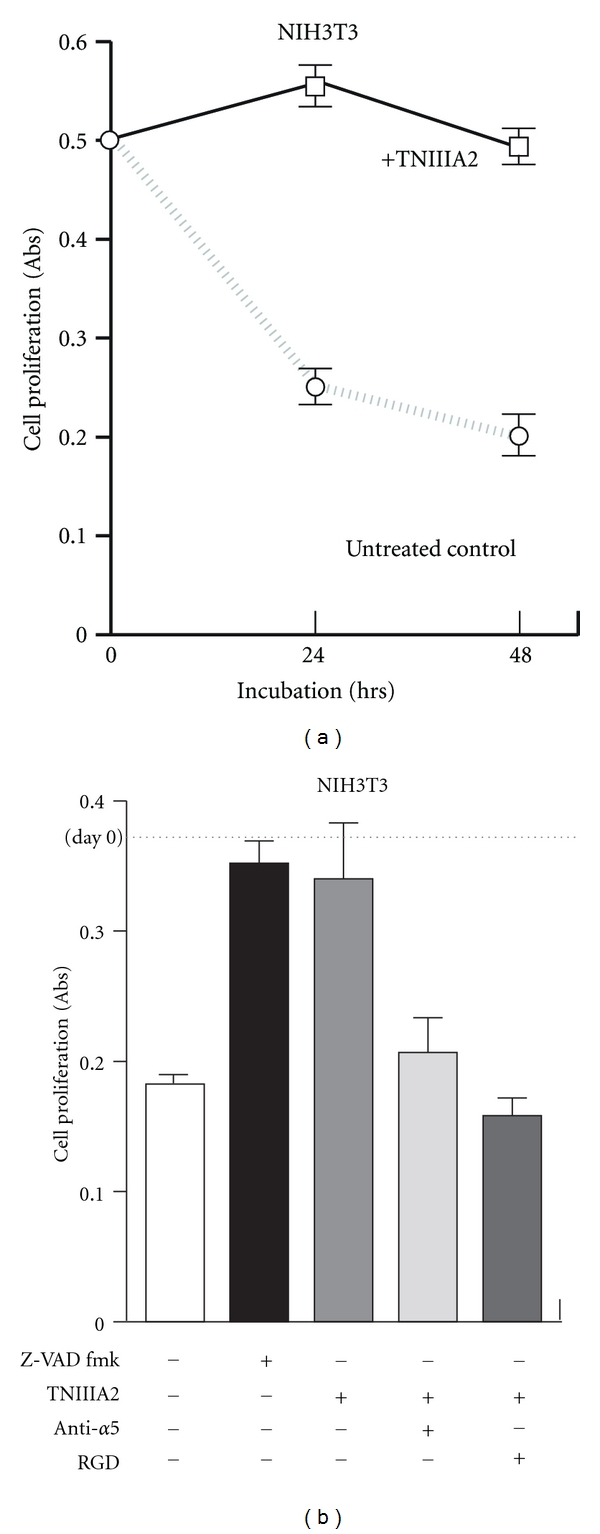
TNIIIA2 protects cells from anoikis by activating the *β*1-integrin. (a) Time-course study of the effect of cell-detachment in survival/proliferation of NIH3T3 cells. (b) Effect of inhibition of apoptosis (Z-VAD) or *α*5*β*1 integrin signal (anti-*α*5 Ab and RGD peptide) in detachment-induced cell death (our unpublished observation).

Recently, we found that detachment-induced cell death, which was repressible by Z-VAD, general caspase inhibitor, was completely blocked by TNIIIA2 administration (Figures 6(a) and 6(b)). This antianoikis effect of TNIIIA2 was abolished by inhibition of *α*5*β*1 integrin (VLA-5) (Figure 6(b)). Activation of Akt and upregulation of Bcl-2 were observed in consistent with inhibition of the detachment-induced cell death by TNIIIA2 (data not shown). These results suggest that TNIIIA2 has a potential to render cells resistant to anoikis.

Platelet-derived growth factor (PDGF) works as a potent mitogen for both untransformed and transformed mesenchymal cells. The binding of PDGF to its receptor PDGFR induces the activation of its intrinsic kinase, which infers activate the Ras/MAP kinase pathway [56]. However, it has also been established that cell proliferation does not occur unless the cells are adhered to the extracellular matrix (ECM) via integrins [57]. Thus, adhesion receptor integrins, as well as growth factor receptors, play an indispensable role in cell proliferation. The collaboration of signaling by integrin ligation with signaling by growth factor receptors is known to enable to amplify the magnitude and duration of activation status in the MAP kinase/ERK pathway.

**Figure 7:**
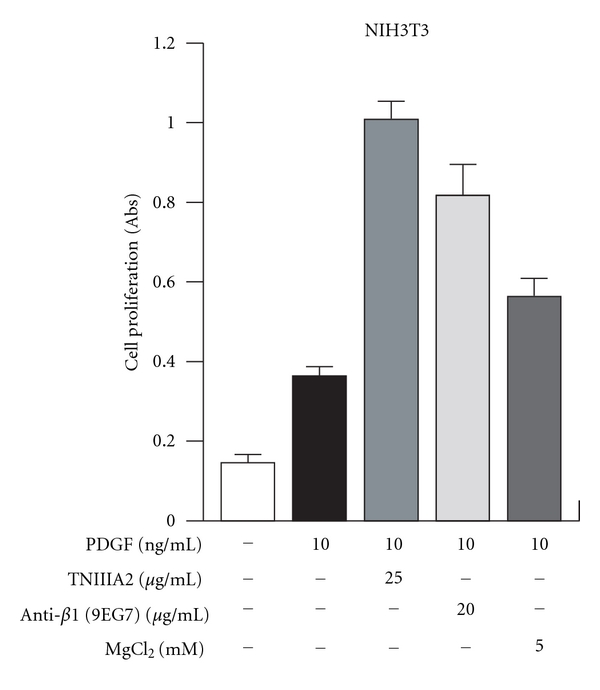
TNIIIA2 accelerates PDGF-induced cell proliferation. PDGF-induced cell growth was enhanced by addition of TNIIIA2, as well as administration with integrin activators (anti-*β*1 and MgCl_2_) (our unpublished observation).

In our investigation, TNIIIA2 showed the ability to accelerate PDGF-induced proliferation of NIH3T3 cell on FN-coated culture dish (Figure 7). Similar to the effect of TNIIIA2, 9EG7, an anti-*β*1 integrin monoclonal antibody, which has the ability to activate *β*1-integrin, also enhanced the PDGF-dependent cell proliferation. Inhibition of *α*5-integrin mediated cell adhesion, but not of *α*4- and *β*3-integrin, could attenuate the effect of TNIIIA2 (unpublished observations), suggesting that stimulation of NIH3T3 cell proliferation by TNIIIA2 is due to activation of *β*1-integrins. In this condition, it was also observed that stimulation of NIH3T3 cell proliferation by TNIIIA2 promotes the autophosphorylation of PDGFR, in which both PDGFR and *β*1-integrin were colocalized in caveolae (data not shown). These observations suggest the existence of crosstalk between ECM signaling and PDGF signaling in cell proliferation. Therefore, the antagonistic drug targeting TNIIIA2-related active site in TN-C molecule, such as anti-TNIIIA2 antibody, might become a new therapeutic drug candidate for diseases relating hyperstimulated cell growth, such as in tumor progression.

## 4. Summary and Future Perspectives

Besides the developing cancer, several researchers recently reported the parallel relationship between TN-C expressions and severity of various diseases, such as chronic liver disease, cardiac infarction, and arthritic joint disease [58–60]. In fact, it has been shown that the ECM proteins, such as TN-C, harbor functional sites within their molecular structure, and these cryptic active sites are disclosed by proteolytic degradation with inflammatory proteinases, including MMPs [5, 61, 62]. Thus, the peptide TNIIIA2 might become a powerful tool for understanding these diseases through the concept “signaling disorder by unusual cell adhesion”.

In the case of cancer progression, we mentioned in this review that TNIIIA2 shows a host-beneficial effect in leukemic situation by inducing apoptosis and/or differentiation. Consistent with our results, it has been reported that the loss of integrin-mediated adhesion resulted in decreased sensitivity to chemotherapy in melanoma [63, 64]. On the other hand, Stupack et al. have reported that unligated integrins trigger apoptotic cell death without any death-inducing signals [65]. Moreover, Ileć et al. have also been reported that integirin-mediated adhesion can promote cell survival although these cells are exposed to stress-associated apoptotic signals [54]. From these facts, we presumed that the ability of TNIIIA2 to induce strong and sustained activation of *β*1-integrins is the key factor in modulating cell survival. We already found a cryptic peptide, FNIII14 from FN, and reported that simultaneous administration of this peptide itself with anticancer drug effectively overcomes CAM-DR of AML [25]. In a series of observations using TNIIIA2, this peptide might be capable for regulating cell survival, growth, and differentiation via controlling cell adhesion to ECM. Since tumor cell is characterized by its immortality, hyper-proliferation, and poor differentiation, there is a possibility that the peptide TNIIIA2 might become a useful therapeutic target for cancer treatment. However, at present, several questions still remain unclear. For example, the regulatory mechanism of TNIIIA2 exposure is not fully explored. Effect of TNIIIA2 or its antagonist* in vivo *should also be tested using tumor transplantation model. Further examinations are expected.

## Figures and Tables

**Table 1:** Expression level of cell adhesion-relating molecules (VLA-4, 5, and syndecan-4) and induced cell adhesion or apoptosis by TNIIIA2 or Mg^2+^ administration in fresh AML cells from patients, peripheral blood cells from healthy adults, and hematopoietic tumor cell lines (the table was modified from [19]).

Cells	Expression (%)			Adhesion		Apoptosis	
	VLA-4	VLA-5	Syndecan-4	+TNIIIA2	+Mg^2+^	+TNIIIA2	+Mg^2+^
*“Fresh AML cells”*							
Patient A	98.2	88.8	48.8	++	++	+	++
Patient B	97.5	98.5	9.5	−	+	−	++
*“Peripheral blood cells”*							
Neutrophil	6.7	N.D.	3.2	−	−	−	−
Monocyte	48.6	N.D.	2.0	−	++	−	++
Lymphocyte	40.5	N.D.	4.4	−	++	−	+
*“Cell lines”*							
B cell							
Ramos	96.5	3.2	92.1	+++	++	+++	++
Raji	94.3	65.3	2.3	−	++	−	++
T cell							
Jurkat	92.8	96.6	42.4	+++	++	+++	++
Erythroid							
K562	9.3	97.2	66.7	+++	++	−	−
Myeloid							
U937	98.1	98.2	87.5	+++	++	++	+++
HL60	99.8	99.7	75.3	+++	++	++	+
THP1	68.5	20.7	30.1	++	++	++	+
THP1 (+PMA)	10.5	18.7	99.2	++	++	−	−

Expression of VLA-4, 5 and syndecan-4 was evaluated by flowcytometric analysis.

## References

[1] Bornstein P, Sage EH (2002). Matricellular proteins: extracellular modulators of cell function. *Current Opinion in Cell Biology*.

[2] Murphy-Ullrich JE (2001). The de-adhesive activity of matricellular proteins: is intermediate cell adhesion an adaptive state?. *Journal of Clinical Investigation*.

[3] Fukai F, Hasebe S, Ueki M (1997). Identification of the anti-adhesive site buried within the heparin-binding domain of fibronectin. *Journal of Biochem*.

[4] Kamiya S, Kato R, Wakabayashi M (2002). Fibronectin peptides derived from two distinct regions stimulate adipocyte differentiation by preventing fibronectin matrix assembly. *Biochemistry*.

[5] Watanabe K, Takahashi H, Habu Y (2000). Interaction with heparin and matrix metalloproteinase 2 cleavage expose a cryptic anti-adhesive site of fibronectin. *Biochemistry*.

[6] Chigaev A, Blenc AM, Braaten JV (2001). Real time analysis of the affinity regulation of *α* 4-integrin: the physiologically activated receptor is intermediate in affinity between resting and Mn2+ or antibody activation. *Journal of Biological Chemistry*.

[7] Saito Y, Imazeki H, Miura S (2007). A peptide derived from tenascin-C induces *β*1 integrin activation through syndecan-4. *Journal of Biological Chemistry*.

[8] Williams DA, Rios M, Stephens C, Patel VP (1991). Fibronectin and VLA-4 in haematopoietic stem cell-microenvironment interactions. *Nature*.

[9] Shain KH, Landowski TH, Dalton WS (2000). The tumor microenvironment as a determinant of cancer cell survival: a possible mechanism for de novo drug resistance. *Current Opinion in Oncology*.

[10] Sachs L (1995). The adventures of a biologist: prenatal diagnosis, hematopoiesis, leukemia, carcinogenesis, and tumor suppression. *Advances in Cancer Research*.

[11] Bradstock KF, Gottlieb DJ (1995). Interaction of acute leukemia cells with the bone marrow microenvironment: implications for control of minimal residual disease. *Leukemia and Lymphoma*.

[12] Hurley RW, McCarthy JB, Verfaillie CM (1995). Direct adhesion to bone marrow stroma via fibronectin receptors inhibits hematopoietic progenitor proliferation. *Journal of Clinical Investigation*.

[13] Molla A, Block MR (2000). Adherence of human erythroleukemia cells inhibits proliferation without inducing differentiation. *Cell Growth and Differentiation*.

[14] Damiano JS, Hazlehurst LA, Dalton WS (2001). Cell adhesion-mediated drug resistance (CAM-DR) protects the K562 chronic myelogenous leukemia cell line from apoptosis induced by BCR/ABL inhibition, cytotoxic drugs, and *γ*-irradiation. *Leukemia*.

[15] Chilosi M, Lestani M, Benedetti A (1993). Constitutive expression of tenascin in T-dependent zones of human lymphoid tissues. *American Journal of Pathology*.

[16] Ocklind G, Talts J, Fassler R, Mattsson A, Ekblom P (1993). Expression of tenascin in developing and adult mouse lymphoid organs. *Journal of Histochemistry and Cytochemistry*.

[17] Atula T, Hedström J, Finne P, Leivo I, Markkanen-Leppänen M, Haglund C (2003). Tenascin-C expression and its prognostic significance in oral and pharyngeal squamous cell carcinoma. *Anticancer Research*.

[18] Leins A, Riva P, Lindstedt R, Davidoff MS, Mehraein P, Weis S (2003). Expression of tenascin-C in various human brain tumors and its relevance for survival in patients with astrocytoma. *Cancer*.

[19] Saito Y, Owaki T, Matsunaga T (2010). Apoptotic death of hematopoietic tumor cells through potentiated and sustained adhesion to fibronectin via VLA-4. *Journal of Biological Chemistry*.

[20] Sugahara H, Kanakura Y, Furitsu T (1994). Induction of programmed cell death in human hematopoietic cell lines by fibronectin via its interaction with very late antigen 5. *Journal of Experimental Medicine*.

[21] Terui Y, Furukawa Y, Sakai T (1996). Up-regulation of VLA-5 expression during monocytic differentiation and its role in negative control of the survival of peripheral blood monocytes. *Journal of Immunology*.

[22] Kapur R, Cooper R, Zhang L, Williams DA (2001). Cross-talk between *α*4*β*1/*α*5*β*1 and c-Kit results in opposing effect on growth and survival of hematopoietic cells via the activation of focal adhesion kinase, mitogen-activated protein kinase, and Akt signaling pathways. *Blood*.

[23] Matsunaga T, Takemoto N, Sato T (2005). Interaction between leukemic-cell VLA-4 and stromal fibronectin is a decisive factor for minimal residual disease of acute myelogenous leukemia. *Nature Medicine*.

[24] Hazlehurst LA, Dalton WS (2001). Mechanisms associated with cell adhesion mediated drug resistance (CAM-DR) in hematopoietic malignancies. *Cancer and Metastasis Reviews*.

[25] Matsunaga T, Fukai F, Miura S (2008). Combination therapy of an anticancer drug with the FNIII14 peptide of fibronectin effectively overcomes cell adhesion-mediated drug resistance of acute myelogenous leukemia. *Leukemia*.

[26] Wang MWJ, Consoli U, Lane CM (1998). Rescue from apoptosis in early (CD34-selected) versus late (Non-CD34-selected) human hematopoietic cells by very late antigen 4- and vascular cell adhesion molecule (VCAM) 1-dependent adhesion to bone marrow stromal cells. *Cell Growth and Differentiation*.

[27] Damiano JS, Cress AE, Hazlehurst LA, Shtil AA, Dalton WS (1999). Cell adhesion mediated drug resistance (CAM-DR): role of integrins and resistance to apoptosis in human myeloma cell lines. *Blood*.

[28] Lévesque JP, Leavesley DI, Niutta S, Vadas M, Simmons PJ (1995). Cytokines increase human hemopoietic cell adhesiveness by activation of very late antigen (VLA)-4 and VLA-5 integrins. *Journal of Experimental Medicine*.

[29] Yamashita Y, Oritani K, Miyoshi EK, Wall R, Bernfield M, Kincade PW (1999). Syndecan-4 is expressed by B lineage lymphocytes and can transmit a signal for formation of dendritic processes. *Journal of Immunology*.

[30] Kaneider NC, Egger P, Dunzendorfer S, Wiedermann CJ (2001). Syndecan-4 as antithrombin receptor of human neutrophils. *Biochemical and Biophysical Research Communications*.

[31] Coulombel L, Vunillet MH, Leroy C, Tchernia G (1988). Linage- and stage-specific adhesion of human hematopoetic progenitor cells to extracellular matrices from marrow fibroblasts. *Blood*.

[32] Williams DA (1994). Molecular analysis of the hematopoietic microenvironment. *Pediatric Research*.

[33] Murti KG, Brown PS, Kumagai MA, Campana D (1996). Molecular interactions between human B-cell progenitors and the bone marrow microenvironment. *Experimental Cell Research*.

[34] Clark BR, Gallagher JT, Dexter TM (1992). Cell adhesion in the stromal regulation of haemopoiesis. *Bailliere’s Clinical Haematology*.

[35] Wilson A, Trumpp A (2006). Bone-marrow haematopoietic-stem-cell niches. *Nature Reviews Immunology*.

[36] Patel VP, Lodish HF (1987). A fibronectin matrix is required for differentiation of murine erythroleukemia cells into reticulocytes. *Journal of Cell Biology*.

[37] Vuillet-Gaugler MH, Breton-Gorius J, Vainchenker W (1990). Loss of attachment to fibronectin with terminal human erythroid differentiation. *Blood*.

[38] Tada T, Widayati DT, Fukuta K (2006). Morphological study of the transition of haematopoietic sites in the developing mouse during the peri-natal period. *Journal of Veterinary Medicine Series C*.

[39] Weinstein R, Riordan MA, Wenc K, Kreczko S, Zhou M, Dainiak N (1989). Dual role of fibronectin in hematopoietic differentiation. *Blood*.

[40] Eshghi S, Vogelezang MG, Hynes RO, Griffith LG, Lodish HF (2007). *α*4*β*1 integrin and erythropoietin mediate temporally distinct steps in erythropoiesis: integrins in red cell development. *Journal of Cell Biology*.

[41] Papayannopoulou T, Priestley GV, Nakamoto B (1998). Anti-VLA4/VCAM-1-induced mobilization requires cooperative signaling through the kit/mkit ligand pathway. *Blood*.

[42] Van Der Loo JCM, Xiao X, McMillin D, Hashino K, Kato I, Williams DA (1998). VLA-5 is expressed by mouse and human long-term repopulating hematopoietic cells and mediates adhesion to extracellular matrix protein fibronectin. *Journal of Clinical Investigation*.

[43] Yanai N, Sekine C, Yagita H, Obinata M (1994). Roles for integrin very late activation antigen-4 in stroma-dependent erythropoiesis. *Blood*.

[44] Hamamura K, Matsuda H, Takeuchi Y, Habu S, Yagita H, Okumura K (1996). A critical role of VLA-4 in erythropoiesis in vivo. *Blood*.

[45] Tanaka R, Owaki T, Kamiya S (2009). VLA-5-mediated adhesion to fibronectin accelerates hemin-stimulated erythroid differentiation of K562 cells through induction of VLA-4 expression. *Journal of Biological Chemistry*.

[46] Elices MJ, Osborn L, Takada Y (1990). VCAM-1 on activated endothelium interacts with the leukocyte integrin VLA-4 at a site distinct from the VLA-4/fibronectin binding site. *Cell*.

[47] Witt O, Sand K, Pekrun A (2000). Butyrate-induced erythroid differentiation of human K562 leukemia cells involves inhibition of ERK and activation of p38 MAP kinase pathways. *Blood*.

[48] Nagata Y, Takahashi N, Davis RJ, Todokoro K (1998). Activation of p38 MAP kinase and JNK but not ERK is required for erythropoietin-induced erythroid differentiation. *Blood*.

[49] Park JI, Choi HS, Jeong JS, Han JY, Kim IH (2001). Involvement of p38 kinase in hydroxyurea-induced differentiation of K562 cells. *Cell Growth and Differentiation*.

[50] Woessmann W, Mivechi NF (2001). Role of ERK activation in growth and erythroid differentiation of K562 cells. *Experimental Cell Research*.

[51] Di Pietro R, Di Giacomo V, Caravatta L, Sancilio S, Rana RA, Cataldi A (2007). Cyclic nucleotide Response Element Binding (CREB) protein activation is involved in K562 erythroleukemia cells differentiation. *Journal of Cellular Biochemistry*.

[52] Seki M, Kameoka J, Takahashi S (2006). Identification of tenascin-C as a key molecule determining stromal cell-dependent erythropoiesis. *Experimental Hematology*.

[53] Siri A, Knauper V, Veirana N, Caocci F, Murphy G, Zardi L (1995). Different susceptibility of small and large human tenascin-C isoforms to degradation by matrix metalloproteinases. *Journal of Biological Chemistry*.

[54] Ilić D, Almeida EAC, Schlaepfer DD, Dazin P, Aizawa S, Damsky CH (1998). Extracellular matrix survival signals transduced by focal adhesion kinase suppress p53-mediated apoptosis. *Journal of Cell Biology*.

[55] Frisch SM, Screaton RA (2001). Anoikis mechanisms. *Current Opinion in Cell Biology*.

[56] Claesson-Welsh L (1994). Platelet-derived growth factor receptor signals. *Journal of Biological Chemistry*.

[57] Chiarugi P (2008). From anchorage dependent proliferation to survival: lessons from redox signalling. *IUBMB Life*.

[58] El-Karef A, Yoshida T, Gabazza EC (2007). Deficiency of tenascin-C attenuates liver fibrosis in immune-mediated chronic hepatitis in mice. *Journal of Pathology*.

[59] Nishioka T, Onishi K, Shimojo N (2010). Tenascin-C may aggravate left ventricular remodeling and function after myocardial infarction in mice. *American Journal of Physiology*.

[60] Midwood K, Sacre S, Piccinini AM (2009). Tenascin-C is an endogenous activator of Toll-like receptor 4 that is essential for maintaining inflammation in arthritic joint disease. *Nature Medicine*.

[61] Sage EH (1997). Pieces of eight: bioactive fragments of extracellular proteins as regulators of angiogenesis. *Trends in Cell Biology*.

[62] Davis GE, Bayless KJ, Davis MJ, Meininger GA (2000). Regulation of tissue injury responses by the exposure of matricryptic sites within extracellular matrix molecules. *American Journal of Pathology*.

[63] Truong T, Sun G, Doorly M, Wang JYJ, Schwartz MA (2003). Modulation of DNA damage-induced apoptosis by cell adhesion is independently mediated by p53 and c-Abl. *Proceedings of the National Academy of Sciences of the United States of America*.

[64] Schwartz MA, McRoberts K, Coyner M (2008). Integrin agonists as adjuvants in chemotherapy for melanoma. *Clinical Cancer Research*.

[65] Stupack DG, Puente XS, Boutsaboualoy S, Storgard CM, Cheresh DA (2001). Apoptosis of adherent cells by recruitment of caspase-8 to unligated integrins. *Journal of Cell Biology*.

